# Transcarotid Artery Revascularization (TCAR): The Best Surgical Intervention Option in an Unusual Presentation of Carotid Dissection in a 37-Year-Old Pregnant Female

**DOI:** 10.7759/cureus.54600

**Published:** 2024-02-21

**Authors:** Herbert Oye, Nitasha Abbas

**Affiliations:** 1 Endovascular and Vascular Surgery, Raleigh General Hospital, Beckley, USA; 2 Endovascular and Vascular Surgery, West Virginia Vascular Institute, Beckley, USA; 3 Medicine, Lincoln Memorial University-DeBusk College of Osteopathic Medicine, Knoxville, USA

**Keywords:** internal carotid artery thrombus, internal carotid artery stenosis, transfemoral carotid angioplasty with stenting, carotid endarterectomy (cea), spontaneous internal carotid artery dissection, transcarotid artery revascularization (tcar)

## Abstract

Carotid artery dissection (CAD) is a condition that compromises blood flow and leads to serious complications such as a stroke or cerebrovascular accident (CVA). This case report demonstrates an unusual case of right internal carotid artery dissection, stenosis of >70%, and an intraluminal thrombus. The patient presented to the emergency department with complaints of right-sided neck pain and severe headache status-post a complicated pregnancy. A computed tomography (CT) angiogram of the right carotid was conducted and showed a right internal carotid artery dissection with 70% luminal stenosis and thrombosis. Carotid endarterectomy (CEA), transfemoral carotid angioplasty with stenting (CAS), or transcarotid artery revascularization (TCAR) were all surgical intervention options that were explored. Risks and benefits were compared between the three surgical intervention options, and transcarotid artery revascularization was deemed the best surgical option in this patient's case.

## Introduction

Carotid artery dissection (CAD) is a condition that occurs when a tear is present in the intimal layer of the carotid artery compromising blood flow [1]. CAD can occur in any age group; however, it is most commonly associated with cerebrovascular accident (CVA) in patients in their early 40s with a higher prevalence in males [1]. Carotid artery dissection is a rare condition but has recently caused 6% of spontaneous carotid dissection in postpartum patients with an association with advanced maternal pregnancy age (>30 years old) [2]. Due to the rarity of this condition, a carotid artery dissection can be commonly missed in these patients until the patient presents with ischemic stroke symptoms or severe neurological deficits [2,3]. Ipsilateral head and neck pain with associated Horner's syndrome or transient ischemic attack are all signs indicating potential carotid artery dissection; however, headache and neck pain are the two most common symptoms present in 60%-90% of cases with postpartum carotid artery dissection [2,3]. Most cases are linked to pregnancy with an increased association with postpartum without any predisposing risk factors [3]. Thus, the best surgical intervention option of performing a carotid endarterectomy (CEA), carotid angioplasty with stenting (CAS), or transcarotid artery revascularization (TCAR) needs to be explored. The study describes a 37-year-old pregnant female with an unusual presentation of right internal carotid artery dissection and highlights the best treatment intervention and its implications.

## Case presentation

**Figure 1:**
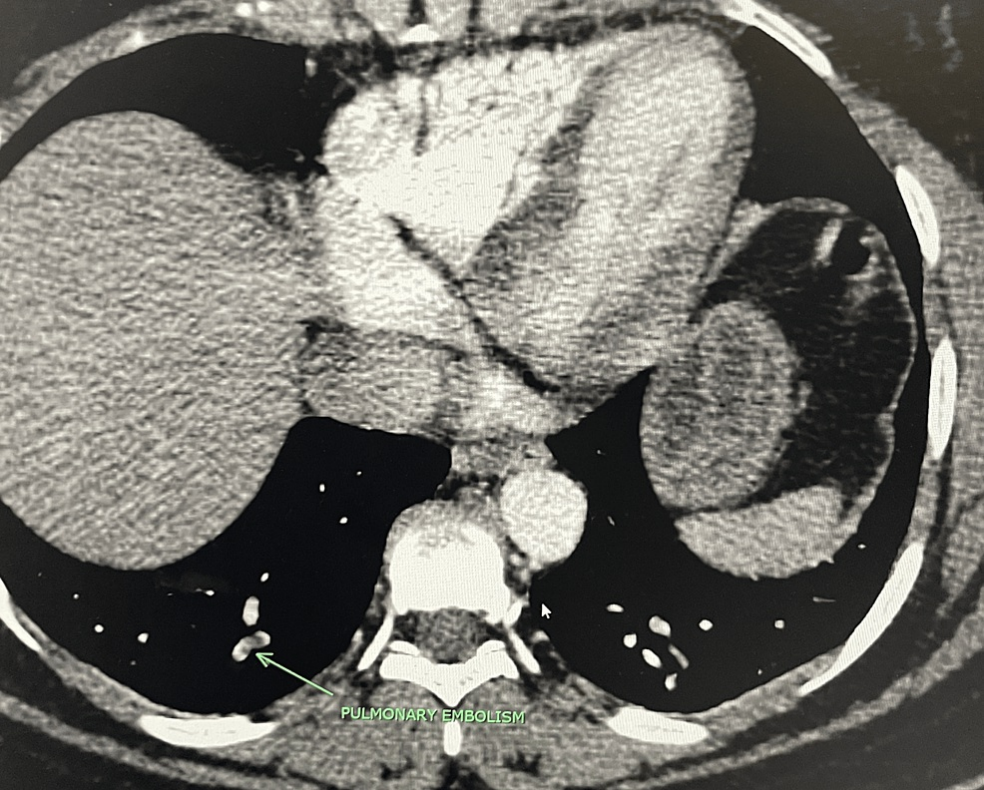
CT of the chest showing right-sided pulmonary emboli (arrow) CT: computed tomography

**Figure 2:**
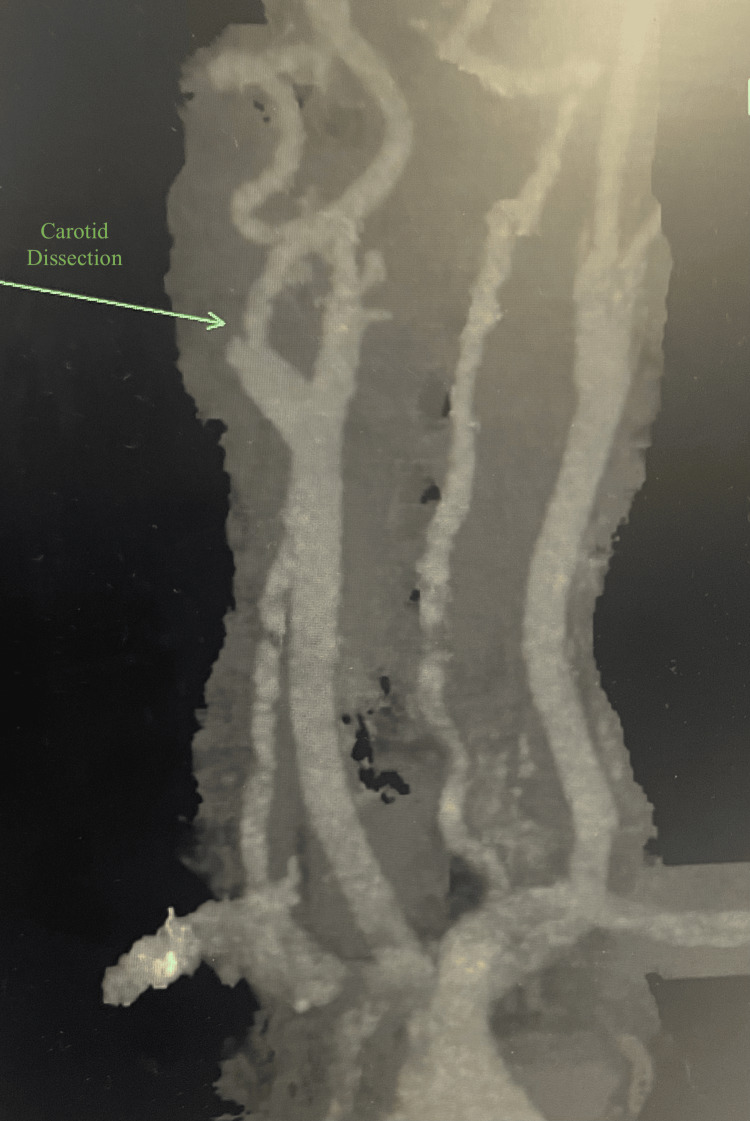
CTA of the neck showing right-sided carotid dissection (arrow) CTA: computed tomography angiography

**Figure 3:**
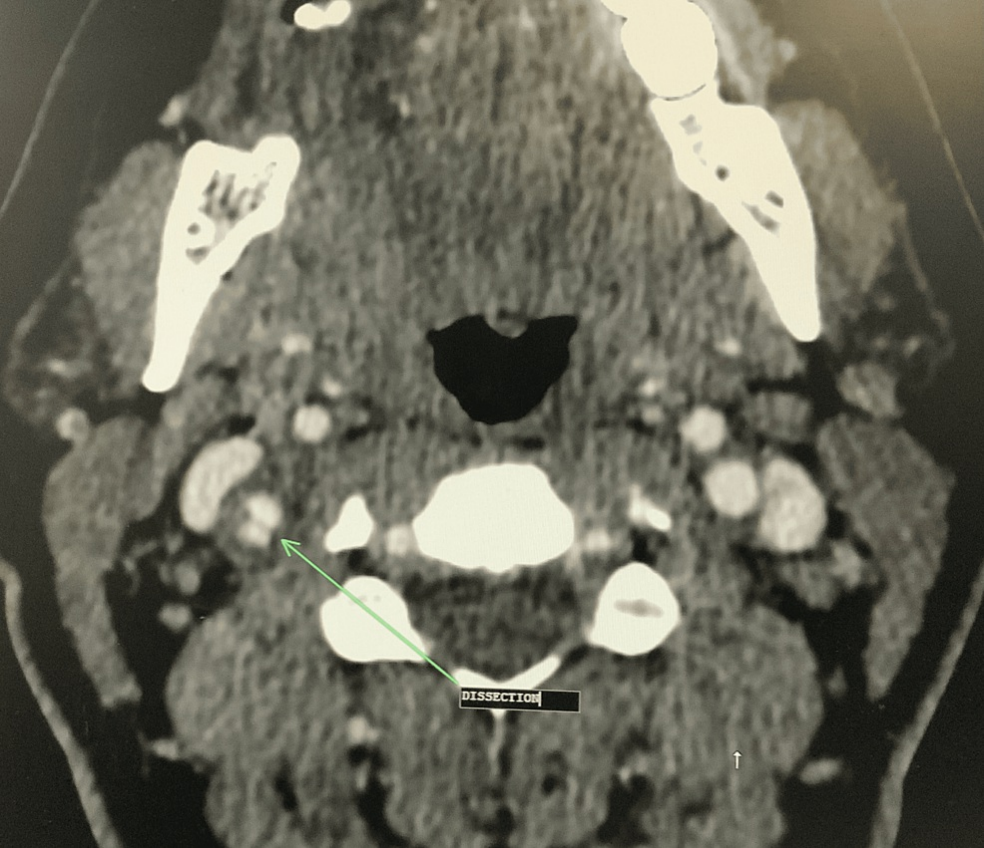
CT of the head showing right-sided carotid dissection (arrow) CT: computed tomography

**Figure 4:**
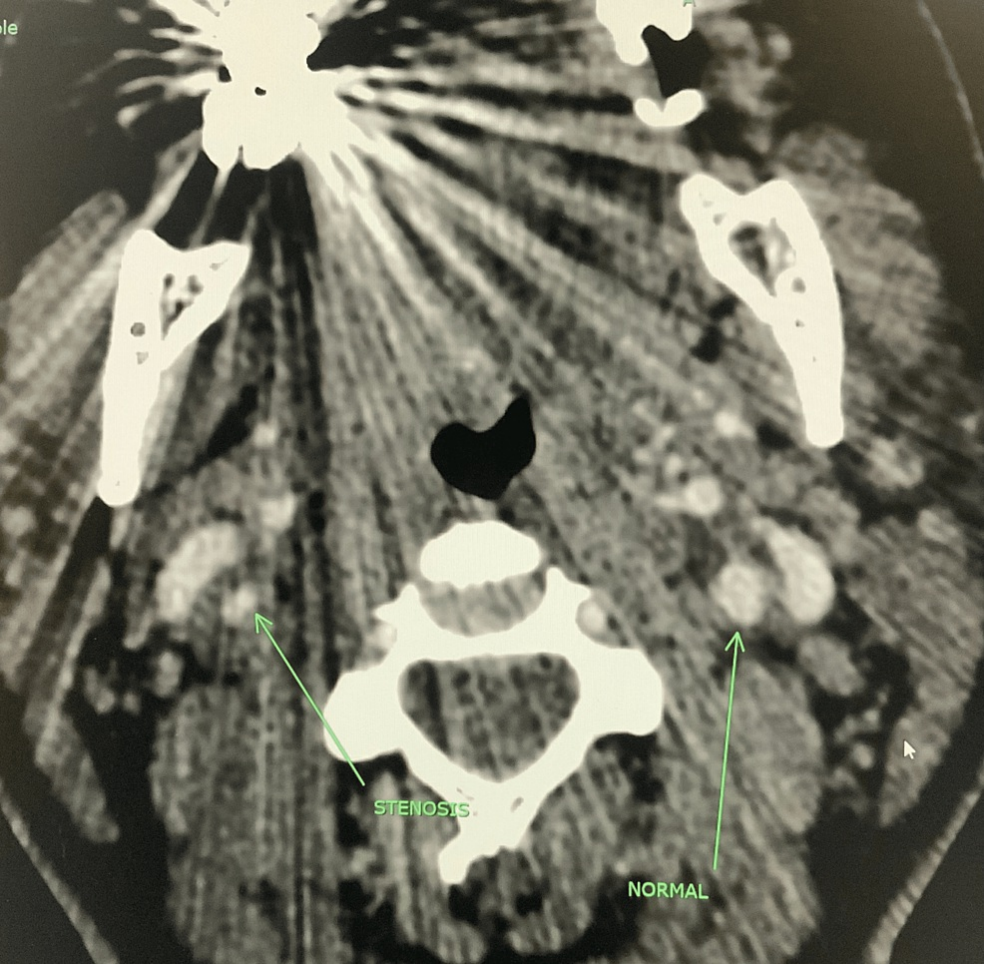
CT of the head showing right-sided (>70%) carotid stenosis and thrombosis (arrow) CT: computed tomography

A 37-year-old pregnant female with no significant history other than hypertension, gestational diabetes, and asthma presented with postpartum hemorrhage, pulmonary embolism, persistent headache, and right-sided neck pain. The patient initially presented to the hospital for a labor induction due to hypertension and potential leaking of amniotic fluid. After a successful vaginal delivery, she experienced severe postpartum vaginal hemorrhage with a loss of ~4,800 cc of blood. She received four units of fresh frozen plasma, four units of packed red blood cells, and one unit of platelet transfusion. Jada, a low-level vacuum intrauterine device, was inserted to control uterine bleeding, and it showed a small amount of bright red blood. After the blood transfusion, the patient was stable and subsequently discharged. She returned to the emergency department several days later with a delayed postpartum hemorrhage. Her postpartum hemorrhage was controlled with an emergency abdominal hysterectomy. She continued to complain of persistent right-sided neck pain and headache accompanied by vertigo. A neurology evaluation was conducted, and she was treated with a migraine cocktail, which consisted of Toradol, Compazine, and Benadryl with minor relief of symptoms. The patient developed episodes of fever, and she was treated with multiple antibiotics, vancomycin, and Zosyn. A computed tomography (CT) scan of the chest was ordered, which showed a small right-sided pulmonary emboli (Figure 1). A CT angiogram of the neck and CT of the head were ordered, which showed right carotid dissection (Figure 2 and Figure 3, respectively) with a luminal compromise of greater than 70% (Figure 4) and an intraluminal thrombus in the right internal carotid artery. Due to the carotid dissection, stenosis, and thrombus, vascular surgery evaluation was initiated.

**Figure 5:**
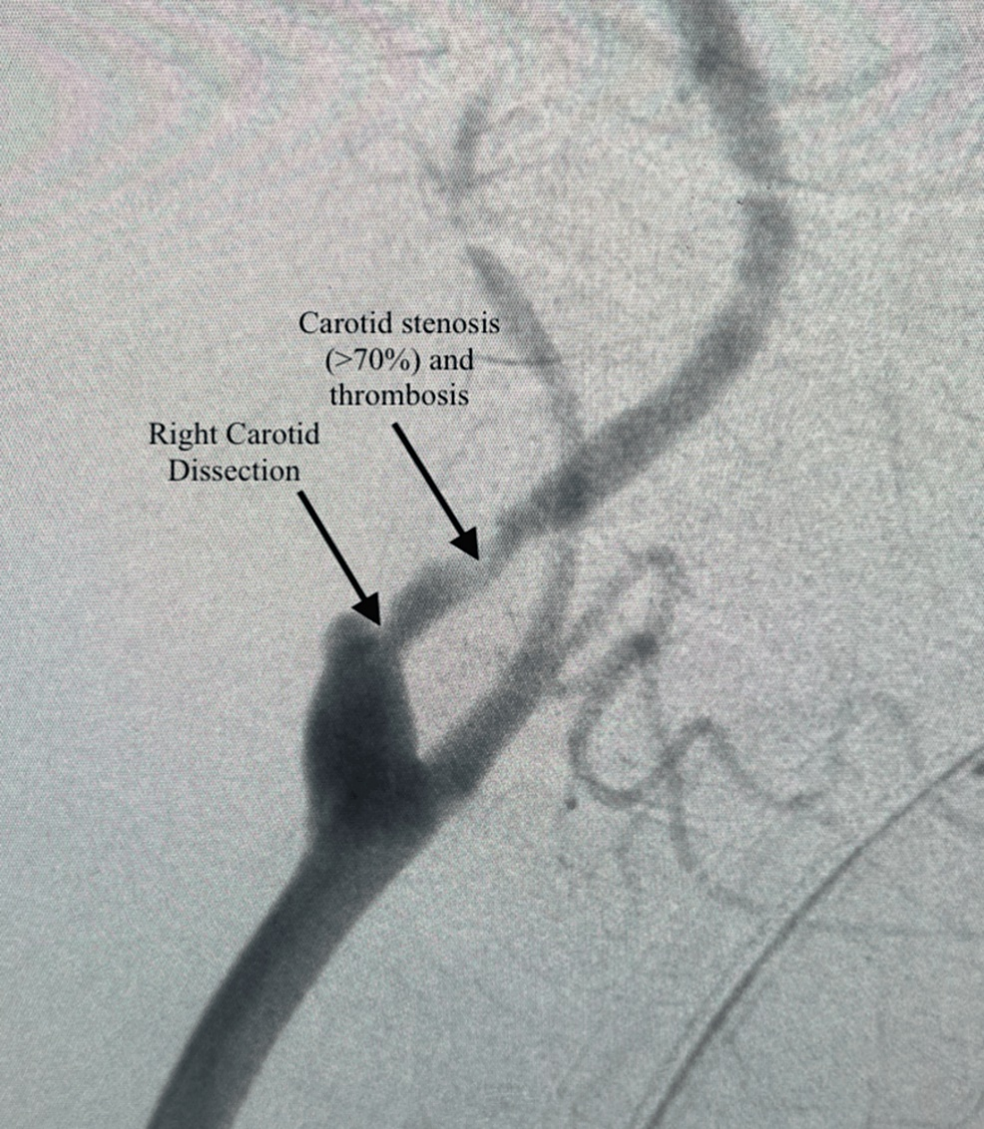
Fluoroscopy of the right internal carotid artery showing internal carotid artery dissection, >70% stenosis, and thrombosis (arrows)

**Figure 6:**
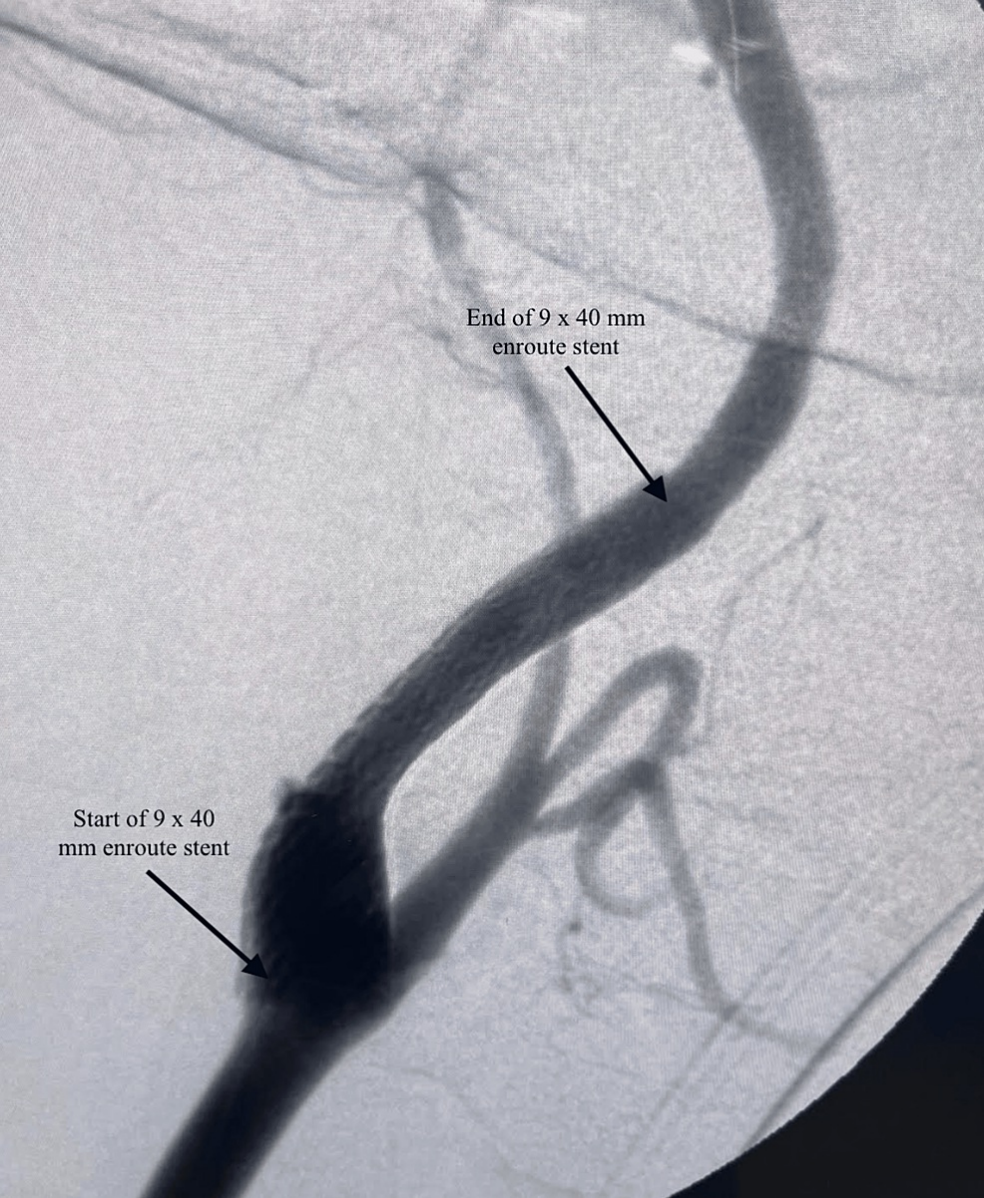
Fluoroscopy of the internal carotid artery status-post right TCAR with stenting using a 9 × 40 mm ENROUTE stent TCAR: transcarotid artery revascularization

Based on the imaging findings, the patient was deemed a good surgical candidate. After exploring various surgical treatment options, it was determined that she was a good fit for transcarotid artery revascularization with stenting due to her history of postpartum hemorrhage, carotid dissection, stenosis, and thrombosis. Informed consent was obtained, and the patient was prepped for surgery. The left common femoral vein was cannulated using a microvenous needle. A microvenous guidewire and sheath were introduced, and an 8-French sheath was placed. A 7-French Silk Road sheath (Silk Road Medical, Inc., Sunnyvale, CA) was placed in the right common carotid artery. Both the arterial and venous sheath were connected to an ENROUTE filter (Silk Road Medical, Inc., Sunnyvale, CA), allowing for the reversal of blood flow from a high arterial to a low venous flow and utilizing the filter to capture emboli. A wire was placed to stop short of the carotid artery stenosis. A CT angiogram with fluoroscopy was performed of the carotid arteries, which demonstrated a right internal carotid artery dissection and right internal carotid artery luminal stenosis (Figure 5) with evidence of thrombosis in the internal carotid artery. We were able to use the microvenous wire, which was a 0.014 guidewire, to cross the lesion into the internal carotid artery with the help of a glide catheter; selective arteriographic studies demonstrated the true lumen and the dissection. Once in the true lumen, a 9 × 40 mm ENROUTE stent was deployed without any complications. The CT angiogram showed excellent flow through the right internal carotid artery (Figure 6). To get the activated clotting time (ACT) to a reasonable number, we had given 12,000 units of heparin throughout the procedure even though she was preloaded with dual antiplatelet therapy consisting of Plavix and aspirin preoperatively as well as Lovenox. Following the procedure, excellent flow was noted, and the clamp was placed. The patient tolerated the procedure well. Post-operation, the patient was stable and was discharged. A week later, the patient presented to the clinic with a resolution of headache.

## Discussion

The patient in this case presented with an unusual case of right internal carotid artery dissection, stenosis of >70%, and an intraluminal thrombus. With these three clinical findings, the patient was deemed a good candidate for surgical intervention. The potential surgical interventions that were explored included a carotid endarterectomy, transfemoral carotid angioplasty with stenting, or transcarotid artery revascularization (TCAR) to determine which surgical intervention would be the best treatment choice for this patient.

For decades, open carotid endarterectomy has been the standard of care for carotid stenosis. Over the past 35 years, carotid angioplasty with stenting has gained ground as another treatment option. More recently, transcarotid revascularization (TCAR), which uses reversal of flow technology, has gained ground and made a significant impact in the management of patients with carotid stenosis. Carotid endarterectomy will be performed with a standard carotid incision or via an eversion technique. The procedure requires meticulous dissection and care to prevent injury to the vagus and hypoglossal nerves. Intraoperative heparin is normally administered, and the patient can be treated with general anesthesia or local anesthesia with monitored anesthesia care. Patients are usually discharged home within 24 hours of the procedure. Common complications associated with carotid endarterectomy include nerve injury with vocal cord paralysis and bleeding or postoperative hematoma. The advantage of carotid endarterectomy is complete plaque removal with either primary closure or patch closure for small caliber vessels. Carotid angioplasty with stenting offers the advantage of minimally invasive intervention, requiring angioplasty and stenting. Increased stroke has been associated with carotid angioplasty with stenting (CAS), despite filters to capture debris during the procedure. This can be attributed to the difficulty in navigating through the aortic arch. TCAR with its flow reversal technology has been linked to a decrease in stroke risk; however, excessive calcification of the carotid artery may result in stent recoil or restenosis. Appropriate patient selection for any of these procedures will be critical for successful outcomes.

​​​​Complicated presentation of internal carotid artery dissection, stenosis, and thrombosis has a high risk of causing a stroke; thus, stroke risks were compared between carotid endarterectomy, transfemoral carotid angioplasty with stenting, and transcarotid artery revascularization. Various trials were compared to better determine the stroke risks between the different surgical procedures. The North American Symptomatic Carotid Endarterectomy Trial (NASCET) discussed the risks, benefits, and effectiveness of carotid endarterectomy in patients with 70%-99% internal carotid artery stenosis. In the trial, 328 patients underwent carotid endarterectomy, and the stroke morbidity/mortality risk was 5.8% [4]. Another retrospective cohort study compared 2,692 patients who underwent a transcarotid artery revascularization procedure and 8,886 patients who underwent carotid endarterectomy; the results showed that the 30-day stroke, death, and myocardial infarction rate was similar, with 3% and 2.6%, respectively [5]. With the comparison between the two trials, it was determined that both carotid endarterectomy and transcarotid artery revascularization had a similar stroke risk.

A carotid endarterectomy is indicated in a symptomatic patient with >60% stenosis of the carotid artery and an asymptomatic patient with >70% stenosis. A carotid endarterectomy could have been performed in a patient with >70% stenosis of the right internal carotid artery; however, in this patient's presentation, a carotid dissection complicated the use of carotid endarterectomy because performing a carotid endarterectomy could further extend the dissection or increase the risk of thrombosis. With these considerations, a carotid endarterectomy was ultimately eliminated as the primary surgical intervention option. The next potential surgical interventions to consider were transfemoral carotid angioplasty with stenting and transcarotid artery revascularization.

A randomized trial discussed that the 30-day death and stroke risk in symptomatic patients who underwent a transfemoral carotid stenting (TF-CAS) was 6%-9% and 2%-4% in asymptomatic patients [5]. Another study compared patients who had a transcarotid artery revascularization procedure to patients who had a transfemoral carotid angioplasty, and the results showed a lower risk of stroke and death in patients with a transcarotid artery revascularization procedure [5]. The stroke risk for TCAR is relatively low at <1%-2% [6]. Overall, transcarotid artery revascularization showed a lower stroke risk, thus favoring the decision to perform a transcarotid artery revascularization over a carotid endarterectomy or transfemoral carotid angioplasty with stenting.

Transcarotid artery revascularization (TCAR) is generally a procedure used in older age patients (>75 years old) or patients with other severe health conditions such as congestive heart failure, myocardial infarction, history of head/neck surgery or radiation, severe pulmonary disease, or uncontrolled diabetes. Although the patient in this case was only 37 years old with no other severe health condition, the patient's history of right internal carotid dissection, stenosis, and thrombosis made her an excellent candidate for transcarotid artery revascularization. Transcarotid artery revascularization uses dynamic blood flow reversal to remove the thrombosis from the right carotid artery, ballooning and stenting the artery in the process to repair the carotid artery dissection and stenosis with cerebral protection.

## Conclusions

Transcarotid artery revascularization (TCAR) with stenting was used as a useful therapeutic option in this young postpartum patient with carotid dissection, stenosis, and thrombosis. The TCAR option of reverse flow and stenting proved unique in addressing the isolated thrombus, dissection, and stenosis. The decision to perform TCAR with stenting helped minimize the risk of additional thrombosis or dissection of the internal carotid artery.
